# Assessment of Oral Health in Long-Term Enteral and Parenteral Nutrition Patients: Significant Aspects of Nursing Care

**DOI:** 10.3390/ijerph20043381

**Published:** 2023-02-15

**Authors:** Sylwia Terech-Skóra, Joanna Kasprzyk-Mazur, Magdalena Leyk-Kolańczak, Agnieszka Kruk, Renata Piotrkowska, Wioletta Mędrzycka-Dąbrowska, Janina Książek

**Affiliations:** 1 Department of Surgical Nursing, Medical University of Gdańsk, 80-211 Gdańsk, Poland; 2 Laboratory of Microbiology, Medical Diagnostic Laboratories Invicta, 81-740 Sopot, Poland; 3 Department of Oncology, Transplantation and General Surgery, University Clinical Centre of Gdańsk, 80-952 Gdańsk, Poland; 4 Department of Anaesthesiology Nursing and Intensive Care, Medical University of Gdańsk, 80-211 Gdańsk, Poland

**Keywords:** oral health assessment, nurses, parenteral nutrition, enteral nutrition

## Abstract

Oral health is an underestimated factor affecting overall human health and quality of life. Long-term enteral or parenteral nutritional treatment requires not only regular assessment of access routes, the patient’s nutritional status, and tolerance to the selected method of nutrition but also of oral health. This article discusses the connections between the influence of chewing function, salivation, and xerostomia on the health of the oral cavity of patients on long-term enteral and parenteral nutrition. In addition, the role of nurses in assessing oral health is presented as well as crucial elements of a comprehensive oral assessment in a nursing care plan. Patients receiving long-term enteral and parenteral nutrition have an increased risk of developing oral diseases. Increasing knowledge about the factors affecting oral health among nurses is crucial to provide appropriate care for patients requiring long-term nutritional treatment with omission of the natural route of food intake. Regular assessment of oral health by nurses should be an important aspect in long-term nutritional treatment recommendations.

## 1. Introduction

The assessment and care of the oral cavity has been an important element in the care of patients in intensive care units. Its effectiveness in preventing ventilation-associated pneumonia (VAP), aspiration pneumonia, or hospital-acquired pneumonia (HAP) has been confirmed by many clinical studies [1]. A recent systematic review and meta-analysis revealed that there is some evidence to confirm that patients with poor oral health have an increased risk of severe COVID-19 infection [2].

Concern for oral health is also very important in the care of patients receiving long-term enteral and parenteral nutrition. Enteral nutrition (EN) therapy has long been recognized as the standard in nutrition treatment and is an invaluable alternative in the case of significant difficulties in naturally consuming food through the mouth. The supply of nutrients to the gastrointestinal tract requires the placement of a feeding tube or catheter or creating a stoma. The greatest benefit of enteral feeding is the maintenance of gut stimulation and integrity [3]. Parenteral nutritional (PN) treatment is primarily used in patients with chronic intestinal failure who suffer from a significant impairment of one of the basic functions: the absorption of essential nutrients from the gastrointestinal tract. Therefore, patients must directly receive proteins, carbohydrates, fats, electrolytes, vitamins, trace elements, and fluids into the bloodstream via a central venous catheter [4]. 

Enteral or parenteral nutritional treatment can be provided both in the short and long terms. The administration of enteral or parenteral nutrition for more than 6 months is defined as long-term medical nutritional therapy [5].

The omission of the natural route of food intake not only deprives the patient of the pleasure of eating but is also associated with the risk of complications and accompanying unpleasant sensations. Impaired masticatory function, regardless of the cause, is an important factor causing oral health problems, and patients, as well as their caregivers, are often unaware of the importance of oral health as an important component of overall health [6]. Lack of regular exogenous mechanical (chewing food) or chemical (taste) stimulation reduces saliva secretion, which leads to prolonged carbohydrate removal, accumulation of food debris and plaque, decrease in saliva pH, and dysbiosis [7]. Moreover, the occurrence of xerostomia may additionally lead to inappropriate oral hygiene behaviors [8]. 

Existing recommendations for long-term enteral and parenteral nutrition address many aspects of nutritional therapy, but none of the documents address oral hygiene or the systematic assessment of oral health [9,10,11]. Systematic oral health assessments and correct diagnoses of oral diseases provide an opportunity for effective treatment at an early stage. Nurse participation in oral health assessment can act as important support in providing comprehensive health care to patients on long-term enteral or parenteral nutrition.

The aim of this study was to raise awareness of the importance of assessing the oral health status of patients on long-term enteral or parenteral nutrition, to highlight the role of the nurse in this regard, and to discuss selected factors such as chewing stimulation, salivary secretion, and xerostomia, which can have a significant impact on oral health in this patient group.

## 2. Oral Health Status in Patients Receiving Long-Term Nutritional Treatment

Oral health is an important part of a person’s physical and mental health. It has been shown to have a significant relationship with many chronic diseases and can affect dietary choices, ability to communicate, and functioning in society [12]. Oral health status is closely related to the functioning of swallowing. The longer enteral or parenteral nutrition is used, the greater the risk of deteriorating oral health status, thus resulting in an impaired swallowing mechanism. In 2022, Drancourt et al. [13] published a systematic review of the literature, searching for associations between oral health status and oropharyngeal dysphagia in people aged over 65 years, including dental status, saliva secretion, and oral motility. They found evidence that a reduction in tongue strength and poor tongue mobility were significantly associated with decreased swallowing ability. It is now known that keeping the masticatory organs, especially the teeth, in good condition has a lifelong impact on overall health. The majority of patients receiving EN are unable to orally take food, so require additional supportive care. The level of oral hygiene in many of these patients is very poor [14]. The duration of enteral feeding implies a deterioration in oral health indicators. In a study carried out at the Outpatient Clinic of Artificial Nutrition in Portugal, patients showed poor oral health before the start of feeding by gastrostomy. During three months of exclusive enteral feeding via percutaneous endoscopic gastrostomy (PEG), all studied oral health indicators, such as mucosal, gingival, dental, and plaque status, deteriorated [6]. 

In the long-term parenteral nutrition patient population, oral health status is of significant importance and can affect overall outcomes. A prospective study with 52 home parenteral nutrition patients revealed that the accumulation of multiple risk factors, such as chronic malabsorption, dehydration, multidrug therapy, and symptoms of xerostomia, combined with nutritional therapy, increased the incidence of dental disease and oral infections. A total of 17 (33%) of the patients experienced a change in their previous pattern of dental attendance, with 14 (27%) attending less frequently and 3 (6%) attending more often [15]. Furthermore, the presence of candida infections of the mouth may have a significant impact on catheter-related bloodstream infection (CRBSI) and pose a serious threat to the health and life of the patient [16]. 

The profile of patients requiring enteral and parenteral nutrition has changed over the years. They tend to be older patients, over 65 years of age, and have serious comorbidities [6,17]. Aging and severe comorbidities affect oral self-care [18]. This condition has been observed by Lopes et al. where 30 out of 40 patients on enteral feeding through PEG experienced difficulties with oral self-care and were dependent on caregiver support for oral care. Considering these changes, the approach to dental care for elderly patients requiring long-term nutritional therapy should take into consideration their health status and self-care capacity [6]. 

## 3. Oral Microflora

The human oral cavity is not a homogeneous ecosystem. The oral microflora begins to form during early childhood and changes throughout life. In the early neonatal period, a large part of the oral microflora comes from the mother, e.g., *Lactobacillus* spp., *Staphylococcus* spp., *Streptococcus* spp., *Bifidobacterium* spp., or Gram-negative rod-shaped bacteria of the *Enterobacterales*. With time, the human bacterial flora begins to stabilize, and its composition depends on exogenous factors, such as living conditions and the type of food and endogenous factors, such as health or genetic conditions [19]. Bacteria included in the oral microflora in adults show a specific topography, colonizing the tongue, teeth, hard palate, gingival pockets, and oral mucosa (Table 1). 

The flora that colonize the oral cavity include both commensal and pathogenic bacteria. Adequate oral hygiene and a balanced diet help to maintain the balance between microorganisms. Changing any factor may create an environment in which the balance of the number of microorganisms is disturbed, which results in the multiplication of pathogenic bacteria. Elderly patients with limited chewing and swallowing ability, ill-fitting dentures, or poor oral hygiene are particularly exposed to oral dysbiosis processes [20].

Oral food intake plays an important role not only in nutrition but also in maintaining the microbiological balance in the complex oral and gut ecosystem. Katagiri et al. observed that the reinitiation of oral nutrition for poststroke patients significantly changed the composition of the oral microbiota, but not that of the gut microbiota. They found that there was a change in the interaction between microbiome species and an increase in the activity of *Lactobacillus salivarius*, which can inhibit the colonization of *Helicobacter pylorii* in the stomach. They suggested that administering *L. salivarius* can prevent dental caries and periodontitis [21]. Parenteral nutrition is one of the factors influencing the composition of the oral microbiota, for example, by reducing the amount of secreted saliva, which, in addition to moisturizing the mucosa, has bacteriostatic and bactericidal effects. Moreover, patients on PN, not consuming food orally or consuming a negligible amount of food, show serious negligence of oral hygiene, resulting from the erroneous impression that oral hygiene is not required if their mouth is not used for food consumption [15]. Microbial changes in the oral cavity that are associated with poor oral hygiene disrupt the intestinal flora balance, which can lead to inflammatory bowel disease (IBD). Additionally, periodontal disease caused by *P. gingivalis* can lead to the destruction of the intestinal barrier and trigger a systemic inflammatory response and aggravate other systemic diseases [22].

## 4. The Importance of Chewing

Chewing is the first and crucial step in digestion and requires the coordination of the tongue, facial muscles, jaw, and teeth. It is an important process not only because of food intake: the masticatory apparatus is significantly related to the process of speech production and has an impact on general health. Chewing stimulates the production of saliva, which indirectly influences digestive functioning [23]. 

In the case of enterally and parenterally fed patients, maintaining the masticatory apparatus in good condition is of great importance. The inability to orally take food or insufficient food intake may result in higher rates of caries, decreased antimicrobial activity of the saliva, drying out of the oral mucosa, adherence of thick sputum to the palate, and neglecting oral hygiene during periods of underlying disease activity [24].

Masticatory performance problems have been associated with loss of teeth, wearing removable dentures, reduced salivation, tongue disease, loss of muscle mass, and increased nutritional deficiencies [25]. Therefore, maintaining the chewing function in good condition is recommended, especially for older patients. Effective chewing does not require oral intake, especially when this is impossible or significantly restricted. Exercises to improve oral muscle strength are simple to perform and consist of chewing exercises with sugar-free chewing gum; clenching, holding, and biting exercises with a chewing aid apparatus; and stretching exercises for the muscles of the face and tongue [18]. Chuhuaicura et al. [26] reported that chewing or sucking a piece of sugar-free mint-flavored gum was associated with an immediate better cognitive performance, improved word memory score, and better spatial memory. An increase in blood flow in the brain was also observed, which may reduce the risk of cognitive impairment.

## 5. The Role and Functions of Saliva

Saliva is a dilute aqueous solution that consists of organic and inorganic components and plays an important role in maintaining the microbiological balance in the mouth. The high water content of saliva helps to moisturize the food and makes it easier to chew. The secretion of saliva is controlled by the autonomic nervous system, and the amount of saliva produced depends on the type and intensity of stimulation. Under physiological conditions, an adult produces 0.5 to 1 L of saliva per day. The main stimulator of salivation is food, and much larger amounts are produced before, during, and after meals. During sleep, physiological saliva secretion is reduced [25]. 

Based on the physiological characteristics of the functioning of the salivary glands, saliva has many important functions, such as protecting the teeth and soft tissues against mechanical, biological, and chemical factors. Continuous moistening of the oral cavity is of key importance for speech and taste and supports the processes of chewing and swallowing [27].

## 6. Xerostomia and Methods of Evaluation

Xerostomia is associated with reduced saliva secretion and a change in its chemical composition, resulting in a dry mouth. The classification includes xerostomia vera (primaria), which results from disturbances in the functioning of the salivary glands and manifests itself as a group of related symptoms; and xerostomia spuria (symptomatica), which is characterized by a subjective perception of dry mouth, without any objective signs of dysfunction of the salivary glands [28]. 

Most people have experienced or experience this feeling every now and then, especially in stressful situations; however, this is usually a short-term discomfort. Chronic xerostomia is a much more serious problem, as it may affect speech, cause swallowing difficulties, taste disturbances, mechanical damage, or chronic oral infections, and result in the need for dentures, affecting the overall quality of life [29]. 

Xerostomia is more commonly reported in people over 65 years of age. The occurrence of xerostomia is strongly associated with comorbidities, polypharmacy and disease treatment, smoking, or inflammation of the salivary glands. Persistent dry mouth may accompany diseases such as type I diabetes, hyperthyroidism, neurosis, schizophrenia, depression, Parkinson’s disease, Alzheimer’s disease, Addison–Biermer anemia, Sjögren’s syndrome, as well as hypercalcemia or avitaminosis (B1, B2, B6, and B12 deficiency). Chronic anxiety and stress also impair the functioning of the salivary glands [30]. 

The long-term use of EN and PN indirectly influences the environment and condition of the oral cavity. Malnutrition and dehydration, common among patients with inflammatory bowel disease or bowel failure after extensive resection of the small intestine, also reduce salivation. Lee et al. [15] found that 81% of respondents experienced discomfort in the mouth during parenteral nutrition therapy, which had a direct impact on the permanent change in the nutritional products additionally taken by mouth in 27% of these patients. Furthermore, another study showed that xerostomia was more common among patients with restricted oral intake, weight loss, and impaired independence [31]. 

Chronic dry mouth has an important psychological aspect, as it significantly affects the quality of life of patients. Therefore, the diagnosis of dry mouth requires a detailed clinical anamnesis, including comorbidities, medications taken, the course of therapies (radiotherapy, dialysis, and nutritional treatment) of the underlying disease, and the assessment of objective symptoms reported by the patient [29]. A summary of the symptoms of xerostomia is presented in Table 2.

More than 100 questionnaires, available in the literature, have been developed for assessing the severity of dry mouth and salivary gland functioning. The most common questionnaires used for the subjective diagnosis of xerostomia are: Xerostomia Inventory (XI), Fox’s Questionnaire, Shortened Xerostomia Inventory (SXI), and Visual Analogue Scale (VAS) [32]. Another test that can be used to visually assess dry mouth is the Challacombe Scale, which describes the 10 characteristic symptoms of the lack of saliva in the mouth. Using the scale, the degree of dryness can be defined as mild, moderate, or severe [33].

## 7. Role of Nursing in Oral Health Assessment and Patient Education

In the recent Global Oral Health Status Report, the WHO highlighted that close to 3.5 billion people worldwide suffer from oral diseases [34]. According to the data of the Ministry of Health from the oral health monitoring programs in the Polish population, in the group of people between 35–44 and 65–74 years of age, the percentage of people with edentulism and periodontal disease is increasing. There is also an increase in the number of people with oral cavity neoplasms. However, the number of dentists in Poland is one of the highest in Europe [35]. Research showed that elderly people are less likely to seek dentist help in solving oral health problems and are particularly vulnerable to poor oral health [36]. Nurses can play an important role in oral assessment because they are a large part of the medical staff, have regular contact with patients, and provide care in hospitals, inpatient care centers, rehabilitation units, and in the community [37]. This is a unique opportunity to educate patients regarding the knowledge and technical skills required in caring for oral health. Involving nurses in interdisciplinary collaboration could be an important supporting strategy and one of the best ways to raise awareness of and promote oral health and improve access to oral health services [38]. 

Updating interprofessional knowledge in the field of oral hygiene is an indispensable element of providing comprehensive nursing care for the patient, as is cooperation with the family in effective prophylaxis.

**Figure 1:**
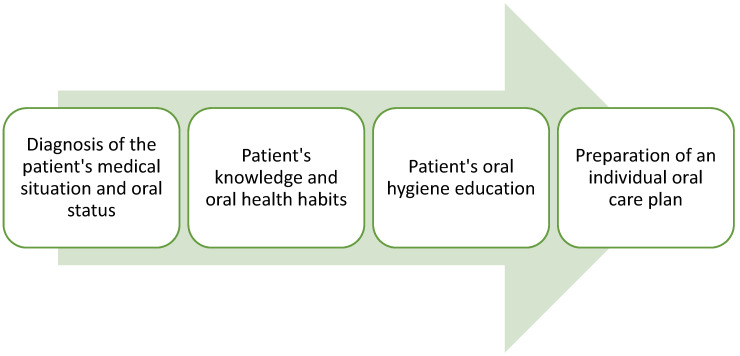
The oral health assessment steps.

The professional preparation of the patient and their caregivers to take action to promote oral health must be based on a comprehensive assessment of the patient’s situation and individual needs. The patient’s oral health assessment steps are shown in Figure 1.

Therefore, the role of specialists other than dentists, such as nurses, is growing in the field of screening and early detection of oral health problems. Kohli et al. [39] reported the full satisfaction of adult patients with the oral cavity assessment performed by qualified nurses. This study also confirmed the value of conducting oral health training, which significantly increased the knowledge of nurses and confidence in conducting oral cavity assessments.

The nursing assessment of oral health should be individualized. This is a very important approach in health care, where patient satisfaction determines the quality of care. Specifically, the nursing care plan for long-term enteral and parenteral nutrition patients should include an oral health assessment, which consists of the following areas and abilities: the patient’s health situation, oral health status, self-care ability, cognitive ability, patient/caregiver knowledge of factors that positively and negatively affect oral health, and preparation of an individualized oral care plan. The assessment categories and objectives are described in Table 3.

## 8. Conclusions

The oral health of patients on long-term enteral and parenteral nutrition requires proper attention. Patients’ ability to maintain their oral health may be limited. The lack of stimulation of the masticatory organ, dry mouth, and inadequate care can cause significant changes in the oral microflora and increase the risk of developing serious diseases in this area. Providing quality oral health care is a major challenge for the healthcare system. A better understanding of the factors affecting the oral health of patients requiring specialized nutritional treatment is elementary in nursing practice. Nurses, in providing care to different groups of patients, play a significant role in educating and promoting oral health. They can perform oral health assessments, train and manage the activities of care assistants, and educate patients and their families. The role of nurses in oral assessment is growing, but is still insufficient. In addition, caring about oral health is still a low priority in society. Nurse education in this area should be promoted, and the priority of care for maintaining oral health in patients with enteral and parenteral nutrition should be raised. 

## 9. Recommendation for Practice

- Comprehensive care for enterally and parenterally fed patients must take into account oral care, especially when disease is active.
- Patients in the hospital and in primary care should be supported by nursing and caring staff trained to accurately recognize symptoms of poor oral health and implement appropriate measures.
- An oral health care assessment should be a standard examination in long-term nutritional therapy.
- The implementation of professional training for nurses in the field of oral cavity assessment and care would improve the activities promoting oral health among adult patients treated with enteral and parenteral nutrition and other groups of chronically ill patients.

## Figures and Tables

**Table 1:** The most common microflora in the oral cavity of adults.

Habitat	Genera/Predominant Bacterium
Lips	*Candida albicans* (corners of the mouth)
	*Micrococcus* spp.
	*Corynebacterium* spp.
	*Neisseria* spp.
	*Staphylococcus* spp.
Tongue	*Streptococcus salivarius*
	*Peptostreptococcus* spp.
	*Veillonella* spp.
	*Actinomyces viscosus*
	*Capnocythophaga* spp.
	*Lactobacillus* spp.
	*Treponema* spp.
	*Fusobacterium* spp.
Buccal mucosa (cheek)	*Actinomyces viscosus*
	*Streptococcus mittis*
	*Capnocytophaga* spp.
	*Fusobacterium* spp.
	*Prevotella intermedia*
Palate	*Actinomyces* spp.
	*Lactobacillus* spp.
	*Streptococcus* spp.
	*Veillonella* spp.
	*Candida* spp.
	*Bacteroides* spp.

**Table 2:** Symptoms of xerostomia.

Type of Symptom	Symptom
Subjective	pain and burning sensation of the oral cavity or on the tongue taste disorder or changed sense of taste difficulty in chewing and swallowing difficulty in speaking loss of appetite difficulty chewing dry and crumbly foods mucosal sensitivity to spicy or acidic foods problem with using complete denture prostheses low quality of life
Objective	weight loss pallor and dry oral mucosa sulcation of the tongue and atrophy of the lingual papillae inflammation or ulcers of buccal mucosa inflammation and fissuring of the lips halitosis (bad breath) dental caries, accumulation of dental plaque, discoloration of teeth oral fungal infections thick, stringy saliva reduced or lack of saliva

**Table 3:** Elements of a comprehensive oral assessment in a nursing care plan.

Assessment Categories	Objectives
Medical interview	General health assessment: systematic diseases, current treatment, medication taken, nutritional status, diet, methods of nutritional treatment, condition.
Oral health examination	Oral assessment: lips, tongue, oral mucosa (color), moisture, integrity, cleanliness, tooth loss or dentures broken, decayed teeth, oral cleanliness, ability to swallow.
Assessment of knowledge and oral care habits	This assessment particular includes the ability to perform oral self-care, knowledge of patients about health problems caused by poor oral hygiene, such as periodontal disease, oral fungal infections, denture care, and the operation of toothbrushes and floss, as well as the patient’s daily oral care habits (time and techniques for brushing and flossing).
Changing or correct oral health behavior	The teaching of oral health includes: physical skills: proper brushing technique, cleaning tongue, flossing technique; selection of oral care products: toothbrushes, mouth rinses, toothpastes, medicated gels, dental floss; recognizing of signs and symptoms of good oral health “look–feel–tell” proper diet and methods of stimulating the masticatory organ.
Individual oral care plan	Assessment oral hygiene depends on: the condition of the patient’s mouth; risk factors for oral health; patient’s and caregivers resources; access to dental services. Assessment of needs and individual oral care plan help maintain a good level of oral hygiene. Note: Regularly evaluate the plan and identify needs.

## Data Availability

Not applicable.

## References

[1] Mędrzycka-Dąbrowska W., Dąbrowski S., Basiński A. (2012). Aktualne zalecenia w pielęgnacji jamy ustnej u pacjentów zaintubowanych i wentylowanych mechanicznie-przegląd piśmiennictwa. Anestezjol. I Ratow..

[2] Qi X., Northridge M.E., Hu M., Wu B. (2022). Oral health conditions and COVID-19: A systematic review and meta-analysis of the current evidence. Aging Health Res..

[3] Kozeniecki M., Fritzshall R. (2015). Enteral nutrition for adults in the hospital setting. Nutr. Clin. Pract..

[4] Lappas B., Patel D., Kumpf V., Adams D., Seidner D. (2018). Parenteral nutrition: Indications, access, and complications. Gastroenterol. Clin. N. Am..

[5] Hadefi A., Arvanitakis M. (2021). How to approach long-term enteral and parenteral nutrition. Gastroenterology.

[6] Lopes S., Tavares V., Mascarenhas P., Lopes M., Cardote C., Godinho C., Oliveira C., Santos C., Oom M., Grillo-Evangelista J. (2022). Oral health status of adult dysphagic patients that undergo endoscopic gastrostomy for long term enteral feeding. Int. J. Environ. Res. Public Health.

[7] Pedersen A., Belstrøm D. (2019). The role of natural salivary defences in maintaining a healthy oral microbiota. J. Dent..

[8] Seo K., Kim H. (2020). Effects of oral health programmes on xerostomia in community-dwelling elderly: A systematic review and meta-analysis. Int. J. Dent. Hyg..

[9] Pironi L., Boeykens K., Bozzetti F., Joly F., Klek S., Lal S., Lichota M., Mühlebach S., Van Gossu A., Wanten G. (2020). ESPEN guideline on home parenteral nutrition. Clin. Nutr..

[10] Bischoff S., Austin P., Boeykens K., Chourdakis M., Cuerda C., Jonkers-Schuitema C., Lichota M., Nyulasi I., Schneider S., Stanga Z. (2020). ESPEN guideline on home enteral nutrition. Clin. Nutr..

[11] Preiser J., Arabi Y., Berger M., Casaer M., McClave S., Montejo-Gonzalez J.C., Peake S., Blaser A., Van den Berghe G., van Zanten A. (2021). A guide to enteral nutrition in intensive care units: 10 expert tips for the daily practice. Crit. Care.

[12] Kisely S. (2016). No mental health without oral health. Can. J. Psychiatry.

[13] Drancourt N., El Osta N., Decerle N., Hennequin M. (2022). Relationship between Oral Health Status and Oropharyngeal Dysphagia in Older People: A Systematic Review. Int. J. Environ. Res. Public Health.

[14] Maeda K., Akagi J. (2014). Oral care may reduce pneumonia in the tube-fed elderly: A preliminary study. Dysphagia.

[15] Lee A., Gabe S., Nightingale J., Burke M. (2012). Intestinal failure and home parenteral nutrition: Implications for oral health and dental care. Clin. Nutr..

[16] Lee A., Gabe S., Nightingale J., Burke M. (2012). Oral health, dental prophylaxis and catheter related bloodstream infections in home parenteral nutrition patients: Results of a UK survey and cohort study. Br. Dent. J..

[17] Folwarski M., Kłęk S., Szlagatys-Sidorkiewicz A., Wyszomirski A., Brzeziński M., Skotnicka M. (2021). Trend observations in home parenteral nutrition. Prevalence, hospitalizations and costs: Results from a Nationwide Analysis of Health Care Provider Data. Nutrients.

[18] Leung K.-M., Chu C.-H. (2023). Dental care for older adults. Int. J. Environ. Res. Public Health.

[19] Graves D., Corrêa J., Silva T. (2019). The oral microbiota is modified by systemic diseases. J. Dent. Res..

[20] Suzuki H., Furuya J., Nakagawa K., Hidaka R., Nakane A., Yoshimi K., Shimizu Y., Saito K., Itsui Y., Tohara H. (2022). Changes in nutrition-intake method and oral health through a multidisciplinary team approach in malnourished older patients admitted to an acute care hospital. Int. J. Environ. Res. Public Health.

[21] Katagiri S., Shiba T., Tohara H., Yamaguchi K., Hara K., Nakagawa K., Komatsu K., Watanabe K., Ohsugi Y., Maekawa S. (2019). Re-initiation of oral food intake following enteral nutrition alters oral and gut microbiota communities. Front. Cell. Infect. Microbiol..

[22] Peng X., Cheng L., You Y., Tang C., Ren B., Li Y., Xu X., Zhou X. (2022). Oral microbiota in human systematic diseases. Int. J. Oral. Sci..

[23] Hollis J. (2018). The effect of mastication on food intake, satiety and body weight. Physiol. Behav..

[24] Olczak-Kowalczyk D., Danko M., Banaś E., Gozdowski D., Popińska K., Krasuska-Sławińska E., Książyk J. (2017). Parenteral nutrition in childhood and consequences for dentition and gingivae. Eur. J. Paediatr. Dent..

[25] Pedersen A., Sørensen C., Proctor G., Carpenter G. (2018). Salivary functions in mastication, taste and textural perception, swallowing and initial digestion. Oral. Dis..

[26] Chuhuaicura P., Dias F., Arias A., Lezcano M., Fuentes R. (2019). Mastication as a protective factor of the cognitive decline in adults: A qualitative systematic review. Int. Dent. J..

[27] Kumar B., Kashyap N., Avinash A., Chevvuri R., Sagar M., Shrikant K. (2017). The composition, function and role of saliva in maintainingoral health: A review. Int. J. Contemp. Dent. Med. Rev..

[28] Tanasiewicz M., Hildebrandt T., Obersztyn I. (2016). Xerostomia of various etiologies: A review of the literaturę. Adv. Clin. Exp. Med..

[29] Villa A., Connell C., Abati S. (2014). Diagnosis and management of xerostomia and hyposalivation. Ther. Clin. Risk Manag..

[30] Anil S., Vellappally S., Hashem M., Preethanath R.S., Patil S., Samaranayake L. (2016). Xerostomia in geriatric patients: A burgeoning global concern. J. Investig. Clin. Dent..

[31] Bressan V., Stevanin S., Bianchi M., Aleo G., Bagnasco A., Sasso L. (2016). The effects of swallowing disorders, dysgeusia, oral mucositis and xerostomia on nutritional status, oral intake and weight loss in head and neck cancer patients: A systematic review. Cancer Treat. Rev..

[32] Khalid A., Elahi S., Qurban A., Atif S. (2022). Xerostomia diagnosis—A narrative review. J. Pak. Dent. Assoc..

[33] Chengappa R., Narayanan V., Khan A., Rakaraddi M., Puttaswamy K., Puttabuddi J. (2016). Utility of two methodologies in the clinical assessment of oral dryness in postmenopausal women. J. Midlife Health.

[34] World Health Organization Oral Health. https://www.who.int/news-room/fact-sheets/detail/oral-health.

[35] Ministerstwo Zdrowia (2016). Programy polityki zdrowotnej. Monitorowanie stanu zdrowia jamy ustnej populacji polskiej na lata. https://www.gov.pl/web/zdrowie/monitorowanie-stanu-zdrowia-jamy-ustnej-populacji-polskiej-w-latach-2016-2020.

[36] Gibney J.M., Wright F.A., D’Souza M., Naganathan V. (2019). Improving the oral health of older people in hospital. Australas. J. Ageing.

[37] Bhagat V., Hoang H., Crocombe L.A., Goldberg L.R. (2020). Incorporating oral health care education in undergraduate nursing curricula - a systematic review. BMC Nurs..

[38] Haresaku S., Uchida S., Aoki H., Akinaga K., Yoshida R., Kubota K., Naito T. (2020). Factors associated with nurses’ performance of oral assessments and dental referrals for hospital inpatients. BMC Oral. Health.

[39] Kohli R., Arora G., Blanc A., Pham E., Gubrud-Howe P. (2021). Oral health clinical training and dental referral program for nurses: An interprofessional collaborative project. Geriatr. Nurs..

